# CRISPR-Cas12a/Aurora Deoxyribozyme Cascade: A Label-Free Ultrasensitive Platform for Rapid *Salmonella* Detection

**DOI:** 10.3390/foods14111892

**Published:** 2025-05-26

**Authors:** Cong Shi, Huimin Tan, Zhou Yu, Weilin Li, Yan Man, Qinghai Zhang

**Affiliations:** 1 Key Laboratory of Environmental Pollution Monitoring and Disease Control, Ministry of Education, School of Public Health, Guizhou Medical University, No. 6 Ankang Road, Guian New Area, Guiyang 561113, China; 15121448276@163.com (C.S.); tanhm3366@163.com (H.T.); yuz2359@163.com (Z.Y.); 18085301797@189.cn (W.L.); 2 Institute of Quality Standard and Testing Technology, Beijing Academy of Agriculture and Forestry Sciences, Beijing 100097, China

**Keywords:** nucleic acid detection, fluorescence sensing, food safety, DNAzyme, pathogen identification

## Abstract

The rapid and ultrasensitive detection of *Salmonella* holds strategic significance for food safety surveillance and public health protection systems. This study innovatively developed a label-free biosensing platform based on the synergistic integration of Clustered Regularly Interspaced Short Palindromic Repeats (CRISPR)-Cas12a and the fluorescent deoxyribozyme Aurora for the efficient detection of foodborne Salmonella. The detection mechanism operates through a molecular cascade reaction: target-activated Cas12a protein specifically degrades Aurora deoxyribozyme via its trans-cleavage activity, thereby abolishing the enzyme’s catalytic capability to convert 4-methylumbelliferyl phosphate (4-MUP) into the highly fluorescent product 4-methylumbelliferone (4-MU). This cascade ultimately enables quantitative target analysis through fluorescence signal attenuation. Following systematic optimization of critical reaction parameters, the biosensing system demonstrated exceptional analytical performance: a detection limit of 1.29 CFU/mL with excellent linearity (R^2^ = 0.992) spanning six orders of magnitude (1.65 × 10^1^–10^6^ CFU/mL), along with high specificity against multiple interfering bacterial strains. Spike-and-recovery tests in complex food matrices (milk, chicken, and lettuce) yielded recoveries of 90.91–99.40% (RSD = 3.55–4.72%), confirming robust practical applicability. Notably, the platform design allows flexible detection of other pathogens through simple replacement of CRISPR guide sequences.

## 1. Introduction

*Salmonella*, as a common foodborne pathogen, poses a persistent threat to human public health safety through animal-derived transmission in the food supply chain [1,2,3]. *Salmonella* infections can lead to acute gastrointestinal disorders (typically diarrhea syndrome and enteric fever), sepsis, and other clinical symptoms, and early diagnosis and timely intervention play a crucial role in improving the prognosis of patients [4,5]. Approximately 9.38 × 10⁷ cases of *Salmonella* infection occur globally each year, resulting in 1.55 × 10⁵ related deaths, creating a significant disease burden in both developing and developed countries [6]. It is therefore important to work together globally to reduce the spread of *Salmonella* through the food supply chain. This emphasizes the critical importance of developing efficient and sensitive rapid *Salmonella* detection technologies for human health and economic development.

As the gold standard for *Salmonella* detection, traditional culture methods (plate culture) offer advantages of high sensitivity and accuracy, but their inherent drawbacks including complicated operations and time-consuming procedures (typically 5–7 days) make them inadequate for modern rapid detection needs [7]. In contrast, polymerase chain reaction (PCR) achieves high sensitivity and specificity through specific primer design, but its dependence on thermal cyclers and requirements for operators with professional backgrounds limit its application in non-central laboratory environments [8]. Considering the application limitations of PCR methods, isothermal nucleic acid amplification (INNA) technology circumvents the thermal cycling steps of traditional PCR through constant temperature reaction systems, eliminating the need for complex thermal cycling equipment, thereby reducing detection equipment costs and demonstrating good potential for field applications [9]. However, its detection system still relies on gel electrophoresis, a complex operation for product analysis, which not only has limited sensitivity but also presents technical bottlenecks such as false positives. Recent advances have shown that the integration of the CRISPR/Cas system with isothermal nucleic acid amplification (INNA) has effectively overcome the key technological bottlenecks of traditional nucleic acid detection methods [10,11,12]. This synergistic strategy not only significantly enhances the molecular recognition specificity and detection sensitivity of the biosensing system, but also achieves compatibility between the reaction temperatures of the two systems, providing unique advantages in nucleic acid detection [13,14,15].

The Clustered Regularly Interspaced Short Palindromic Repeats (CRISPR) system is an adaptive immune mechanism evolved in prokaryotes (bacteria and archaea). This system employs a programmable sequence-targeted recognition module to achieve precise identification and cleavage of exogenous nucleic acids. It specifically targets invading genetic elements, including bacteriophage genomes and plasmid DNA, through its RNA-guided nuclease activity. Beyond establishing robust immune protection against viral infections, this precise nucleic acid recognition mechanism has fundamentally transformed the development of next-generation biosensing technologies [16,17]. When Cas12a, guided by crRNA, specifically recognizes double-stranded target DNA (T-rich PAM site), the RuvC domain of Cas12a cleaves dsDNA generating sticky ends, while Cas12a with trans-cleavage properties indiscriminately cleaves nearby non-targeted ssDNA [18]. This unique trans-cleavage characteristic makes it a powerful signal amplifier, perfectly combining target recognition and signal amplification functions [19,20,21]. Currently, the mainstream structure of Cas12a reporters adopts dual-labeled (FAM fluorophore/BHQ1 quencher) ssDNA to detect ssDNA, achieving target quantification through fluorescence recovery caused by enzymatic cleavage [20]. Although this configuration is simple and efficient, the requirement for dual labeling significantly increases its cost. Therefore, developing a label-free, highly sensitive, and highly specific Cas12a reporter fluorescence detection method has enormous application value.

DNAzymes are biosensing elements that combine enzymatic catalytic capabilities with the programmability of nucleic acid molecules. They offer multiple advantages for biosensing applications [22,23]. First, they can be mass-produced at low cost through scalable chemical synthesis processes. Second, they remain stable in complex biological environments, including nuclease-rich matrices, without inactivation. Third, they typically undergo denaturation and refolding processes without losing functionality. Finally, their properties can be precisely engineered through artificial evolutionary platforms.

DNA enzyme-based fluorescent signal generation strategies have been widely reported in the field of bioassays. For example, Nakayama et al. [24] developed a G-quadruplex (G4) fluorescent substrate system. This approach utilizes complexes ligated with hemin to catalyze fluorescent substrates such as Amplex Red in an H_2_O_2_-containing environment. This system has shown potential for detecting tumor markers. However, one significant limitation exists: hemin itself has inherent peroxidase activity. This intrinsic activity, particularly in the presence of H_2_O_2_, catalyzes the oxidation of the substrate to generate a signal [25]. Consequently, this results in higher background signals, which compromises detection sensitivity. Recently, Volek et al. [22] successfully identified a new DNAzyme named “Aurora”, which operates under mild conditions, has a small structure, requires no labeling, can be synthesized rapidly at low cost, and is an efficient fluorescence enhancer. With its significant advantages of high specificity and low background, Aurora demonstrates extraordinary potential in biosensing applications; however, its potential application in CRISPR systems has not yet been thoroughly explored.

The main objective of this study was to develop a label-free biosensing platform based on the synergistic integration of CRISPR-Cas12a and the fluorescent deoxyribozyme Aurora for rapid and sensitive detection of foodborne *Salmonella*. This platform features an innovative molecular cascade reaction system that activates Cas12a’s specific cleavage capability when target pathogen DNA is present, thereby regulating Aurora enzyme activity and enabling quantitative analysis through fluorescence signal attenuation. This strategy innovatively addresses the limitation of traditional CRISPR detection systems that rely on fluorescently labeled probes, providing new insights for developing simple, economical, and highly sensitive pathogen detection technologies.

## 2. Materials and Methods

### 2.1. Materials and Reagents

All DNA and RNA oligonucleotides purified by HPLC (see Table S1 for details of specific sequences) were chemically synthesized by Sangon Biotech Co., Ltd. (Shanghai, China). Bacterial strains used in the experiments included *Salmonella typhimurium* (ATCC 14028), *Bacillus cereus* (ATCC 15816), *Staphylococcus aureus* (ATCC 25923), *Escherichia coli O157:H7* (CICC 21530), and methicillin-resistant *Staphylococcus aureus* (ATCC 43300), which were commercially purchased from the American Model Culture Collection and Repository (ATCC, Manassas, VA, USA) and China Industrial Microbial Strain Collection and Management Centre (CICC, Beijing, China). LbCas12a was purchased from NEW ENGLAND BioLabs (Ipswich, MA, USA). Recombinase polymerase amplification (RPA) reaction was carried out using TwistAmp^TM^ Basic Kit (TwistDXTM, Cambridge, UK). Bacterial gDNA isolation kit was purchased from BIOMIGA, Inc. (San Diego, CA, USA). Loading buffer was purchased from Tiangen Biotech Co., Ltd. (Beijing, China). Sangon Biotech Co., Ltd. (Shanghai, China) provided the nucleic acid dye 4S-GelRed, 4-methylumbelliferyl phosphate (4-MUP), SanPrep nucleic acid purification column, and DNA marker. Routine chemical reagents (including buffer components) were prepared using analytically pure-grade products provided by Beijing Chemical Reagent (Beijing, China).

### 2.2. Bacterial Culture and Extraction

All strains were inoculated in Brain Heart Infusion (BHI) broth medium and incubated in an electrically heated thermostatic incubator (Tianjing Taisote Medical Equipment, Tianjin, China) at 37 °C for 24 h. In order to quantitatively analyze the bacterial concentration, the bacterial stock solution was serially diluted in a 10-fold gradient, and bacterial dilutions with a concentration gradient of 1:10^0^ to 1:10^9^ were prepared. A total of 100 μL of each gradient dilution was uniformly spread on the surface of agar plates, and then the inoculated plates were incubated in a constant temperature incubator at 37 °C for 24 h to determine the concentration of live bacteria in the bacterial stock solution by plate counting method (Figure S1). Bacterial DNA was extracted using a bacterial gDNA isolation kit and stored at −20 °C for subsequent use.

### 2.3. RPA Amplification and Validation

RPA amplification of invA gene was performed using forward primer (TGTTGTCTTCTCTATTGTCACCGTGGTCCAG) and reverse primer (CATCTGTTTACCGGGCATACCATCCAGAGAAAA). The isothermal amplification reaction system contained 2.4 μL of each forward/reverse primer at a working concentration of 10 μM, 2.5 μL of MgOAc (280 mM), 29.5 μL of Primer Free Rehydration buffer, 1 μL of target template, and 12.2 μL of ultrapure water, in a total volume of 50 μL. All reaction components were prepared on ice and quickly transferred to a Thermal Cycler (Bio-Rad Laboratories, Hercules, CA, USA) and incubated at 39 °C for 20 min. The RPA products were purified using a Sanpred Spin Column & Collection Tube, and the purified RPA products were analyzed by 2% (*w/v*) agarose gel electrophoresis with 1× TAE as electrophoresis buffer. The electrophoresis apparatus (LiuYi Instrument, Beijing, China) was set to a constant voltage of 110 V, and the run time was 45 min. After electrophoresis, the results were visualized and analyzed by a gel imaging system (Bio-Rad Laboratories, Hercules, CA, USA).

### 2.4. Label-Free Detection of Salmonella by Aurora-Based CRISPR/Cas12a Sensor

The biosensing detection system consisted of two systems, A (CRISPR-Cas12a detection system) and B (fluorescence signal amplification system). System A was performed by adding different concentration gradients of RPA amplicon (30 μL), crRNA (5 μL, 200 nM), Cas12a (5 μL, 200 nM), reaction buffer (4 μL, 50 mM Tris-HCl, 250 mM NaCl, 50 mM MgCl_2_, pH 7.4), and Aurora (6 μL, 100 μM). The cells were vortexed and shaken by a vortex oscillator (Shanghai Bowen Instrument, Shanghai, China) and subsequently incubated in an electrically heated thermostatic incubator for 30 min at 37 °C, to activate the trans-cleavage activity of Cas12a.

To prepare system B, another EP tube was taken to prepare 50 μL of reaction solution containing KCl (10 μL, 2000 mM), ZnCl_2_ (10 μL, 10 mM), Tris-HCl reaction buffer (10 μL, 50 mM Tris-HCl, pH 7.4), DMSO (10 μL, 50% *(v*/*v*)), and 4-MUP (10 μL, 600 μM).

The pre-incubated system A reaction solution was mixed with system B reaction solution in equal volume (total volume 100 μL), and the reaction was allowed to stand at room temperature for 30 min to allow the full reaction of Aurora with 4-MUP. All measurements were performed at least three times. The fluorescence intensity was measured using a Multimode Plate Reader EnVision^®^ (Perkin Elmer, Wellesley, MA, USA) at an excitation wavelength of 360 nm and an emission wavelength of 448 nm, and error bars were generated by calculating the standard deviation of three replicate experiments.

### 2.5. Preparation of Spiked Samples

Milk (whole milk, 3.2% protein, 3.6% fat, pasteurized), chicken (fresh chicken breast, skinless), and lettuce (loose-leaf lettuce, conventionally grown) were purchased from a local supermarket (Beijing, China). The chicken and vegetable samples (25 g each) were weighed using an electronic balance (LABGIC, Beijing, China). Each sample was then placed into a separate sterile homogenizing bag containing 225 mL of Tris-HCl solution. The samples were homogenized and stirred for 1 min with a blender to prepare 1:10 homogenates. These homogenates were stored at 4 °C until further use. Milk samples (250 mL) were separated by high-speed centrifugation (Eppendorf, Hamburg, Germany) at 8050× *g* for 10 min. This process removed the upper fat layer and the lower precipitate layer. The intermediate liquid phase was carefully aspirated and then filtered through an ultrafiltration membrane. The filtered solution was diluted 10-fold and stored at 4 °C until use.

## 3. Results and Discussion

### 3.1. Novel Strategy for Highly Sensitive Detection of Salmonella typhimurium Based on Synergistic Action of CRISPR-Cas12a and Aurora Fluorescence System

The novel fluorescent enzyme Aurora exhibits unique autophosphorylation properties, effectively catalyzing the transfer of phosphate groups (P) from the 4-MUP substrate to its own 5′ terminus. This biochemical process facilitates the dephosphorylation of the 4-MUP molecule, subsequently generating the highly fluorescent product 4-methylumbelliferone(4-MU) [22]. The mechanism underlying our CRISPR-Cas12a biosensing detection system operates through molecular cascade reactions: when the invA gene from *Salmonella typhimurium* is present, the Cas12a-crRNA complex binds specifically to target DNA sequences, triggering both cis-cleavage of the target DNA and trans-cleavage activity toward nearby single-stranded DNA. This trans-cleavage activity causes degradation of Aurora enzymes, thereby inhibiting their catalytic function in the dephosphorylation of 4-MUP. Consequently, the fluorescence intensity decreases in proportion to the target DNA concentration, creating a quantifiable inverse relationship between signal output and target presence. This mechanistic integration of CRISPR-Cas12a’s precise recognition capability with Aurora’s fluorescent signal generation establishes a highly sensitive label-free detection platform for *Salmonella typhimurium*.

**Figure 1:**
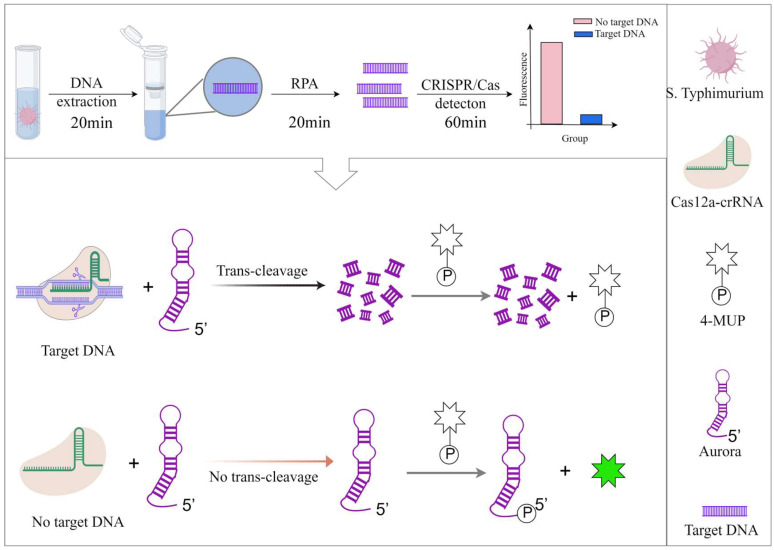
Schematic illustration of the working principle of a highly sensitive biosensor for Salmonella detection based on the CRISPR-Cas12a/Aurora system (Created in Figdraw. Cong Shi (2025) https://www.figdraw.com).

As shown in Figure 1, the entire detection workflow begins with the specific amplification of the invA gene from *Salmonella typhimurium* DNA using recombinase polymerase amplification (RPA) technology. The amplification product then activates the dual cleavage function of the CRISPR-Cas12a system. The cis-cleavage activity of the system precisely recognizes and cleaves the target double-stranded DNA, while simultaneously triggering the trans-cleavage activity, which non-specifically cleaves single-stranded DNA in the surrounding environment [26]. When a target sequence is present, Cas12a-mediated single-stranded DNA cleavage causes Aurora to break. This change results in Aurora losing its ability to bind 4-MUP substrates, thereby significantly reducing the fluorescent signal output of the system. In contrast, if the target sequence is not present, Cas12a remains inactive. At this point, Aurora maintains its intact catalytic function and continuously converts 4-MUP to the fluorescent product 4-MU, generating a strong fluorescent signal output.

### 3.2. Comprehensive Validation of the CRISPR-Cas12a/Aurora-Based Detection System for Salmonella typhimurium

**Figure 2:**
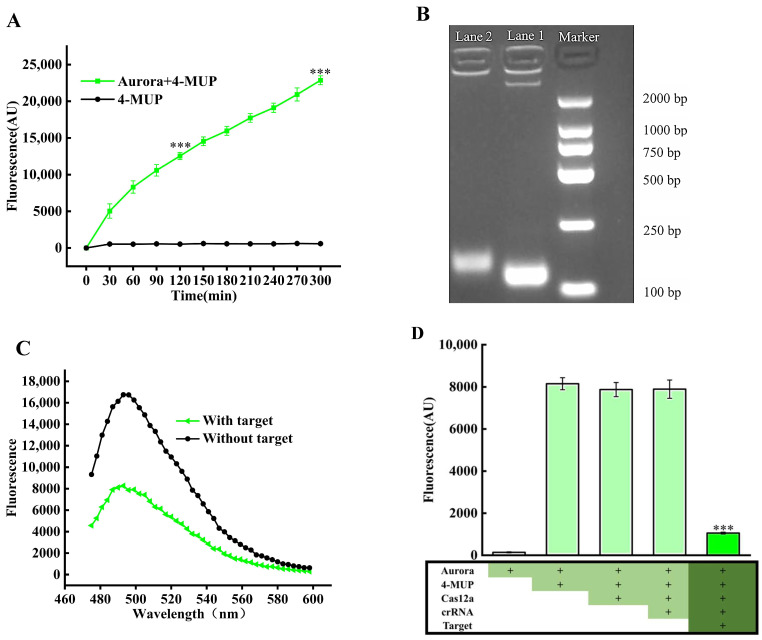
Comprehensive validation of the detection methodology. (**A**) Characterization of Aurora-mediated fluorescence signal generation and stability. (**B**) Electrophoretic analysis confirming the specificity of RPA-amplified invA gene products. (**C**) Evaluation of RPA amplification products’ capability to activate CRISPR-Cas12a trans-cleavage activity using G4 dimer/ThT fluorescence reporter system. (**D**) Assessment of CRISPR-Cas12a system efficiency in cleaving Aurora enzyme under various experimental conditions. *** *p* < 0.001, Student’s unpaired *t*-test.

To comprehensively evaluate Aurora’s performance in terms of catalytic activity, stability, and specificity toward the 4-MUP substrate, this study systematically monitored the dynamic changes in fluorescence intensity of the reaction system over a 0–300 min period, as illustrated in Figure 2A. As the reaction progressed, the fluorescence signal exhibited a distinct phasic enhancement pattern: during the initial 0-120 min, the signal increased rapidly (F/F_0_ = 23.95 ± 0.59) (*p* < 0.001); after 120 min, the growth rate significantly decelerated but continued to rise, reaching a remarkable 39.62 ± 2.73-fold increase (F/F_0_) at 300 min (*p* < 0.001) (where F represents fluorescence measurements under certain conditions and F_0_ represents initial fluorescence measurements). Notably, in the control group containing only 4-MUP, no significant fluorescence signal variations were observed throughout the monitoring period, an experimental phenomenon that mainly stems from the extremely slow rate of spontaneous decomposition of 4-MUP, which produces almost no detectable fluorescence signal change. This feature effectively avoids the interference of background signals generated by non-enzymatic reactions, enabling us to clearly determine that the observed fluorescence signal changes originate from the catalytic reaction of Aurora rather than from the spontaneous decomposition of 4-MUP, thus effectively eliminating potential false-negative signals that might arise from the spontaneous decomposition of 4-MUP. More importantly, the 4-MU generated through Aurora catalysis exhibited stable fluorescence characteristics during 300 min of continuous observation, without any obvious decay trend, indicating that the generated fluorescent product 4-MU possesses a stable chemical structure. This property provides a reliable signal foundation for the CRISPR-Cas12a detection system and effectively prevents the risk of false positives due to unstable fluorescence signals.

To rigorously validate the specificity of RPA amplification products, target DNA was extracted from samples, subjected to RPA amplification, purified through purification columns, and analyzed via agarose gel electrophoresis. As shown in Figure 2B, lane 2 (*Salmonella typhimurium* DNA amplification product) revealed a distinct single band of 120 bp, precisely matching the theoretical expectation, which strongly confirmed that RPA amplification successfully and specifically targeted the signature invA gene of Salmonella typhimurium and generated high-purity amplification products. Concurrently, lane 3 (positive control) exhibited equally clear and well-defined band signals, further verifying the reliability and reproducibility of the entire experimental system. The high specificity of RPA for invA gene amplification demonstrates advantages over traditional PCR methods, which typically require more stringent temperature control and longer amplification times [13]. These experimental data collectively demonstrate that the established RPA amplification system exhibits excellent specificity for *Salmonella typhimurium* DNA recognition, establishing a solid technical foundation for subsequent highly sensitive detection based on the CRISPR-Cas12a platform.

Subsequently, a G-quadruplex dimer (G4 dimer)/thioflavin T (ThT) fluorescence reporting system was employed to systematically validate the capacity of RPA amplification products to activate the CRISPR-Cas12a system. G4 dimer/ThT exhibits a 9-fold increase in fluorescence intensity compared to G4/ThT [27]. As illustrated in Figure 2C, experimental results revealed a significant difference in fluorescence intensity between the experimental group (containing target DNA) and the control group (without target DNA). This distinction has a clear molecular basis. The Cas12a protein in the experimental group becomes activated after specifically recognizing the target DNA. Once activated, it effectively degrades the G4 dimer through its trans-cleavage activity. This degradation interferes with ThT binding to the G4 dimer. The result is a marked attenuation of the fluorescence signal. In contrast, fluorescence intensity in the control group consistently maintained at elevated levels (peak approximately 16,000 fluorescence units), demonstrating that in the absence of target DNA, Cas12a protein remains in an inactive state, unable to cleave the G4 dimer, thus allowing ThT to stably bind with intact G4 dimer and generate intense fluorescence signals. This series of experimental findings robustly confirm that the RPA-amplified invA gene fragments can highly specifically activate the trans-cleavage activity of Cas12a protein, establishing a critical molecular foundation for the subsequent detection system based on this principle.

Additionally, we employed native PAGE (polyacrylamide gel electrophoresis) to investigate the cleavage activity of activated Cas12a against Aurora substrates. As shown in Figure S3, in lane 1, we added only the Aurora enzyme as a reference, clearly revealing the characteristic bands of its intact structure (highlighted in the red dashed box). In lane 2, when Aurora was co-incubated with the activated Cas12a-crRNA-target DNA complex, the characteristic Aurora bands were significantly weakened, while multiple smaller fragments with higher migration rates appeared at the bottom of the gel (highlighted in the blue dashed box), directly demonstrating that Aurora had been effectively degraded into smaller DNA fragments by Cas12a’s trans-cleavage activity. Lane 3 displays the migration pattern of the Cas12a-crRNA complex with target DNA alone, while lane 4 serves as a control containing only target DNA, with these control groups helping to clearly distinguish the positions of each component in the gel. From the schematic diagram on the left side of the gel, one can intuitively observe the process of Aurora being cleaved from its intact structure (upper) into multiple smaller fragments (lower). These electrophoretic results provide direct and compelling molecular-level evidence, clearly confirming our designed detection mechanism in which activated Cas12a efficiently cleaves Aurora DNAzyme molecules, thereby providing a solid experimental foundation for the working principle of the entire detection system.

Finally, this study conducted an in-depth assessment of the CRISPR-Cas12a system’s ability to cleave the Aurora enzyme. As illustrated in Figure 2D, the experimental results demonstrated a striking contrast effect: the addition of 4-MUP, Cas12a, and crRNA to Aurora, either individually or in various combinations, had no effect on its catalytic activity. Only when 4-MUP, Cas12a, crRNA, and target DNA were simultaneously present did we observe a decrease in fluorescence production, indicating a direct relationship between the change in fluorescence signal and the addition of target DNA (*p* < 0.001). This differential phenomenon can be explained through the following molecular mechanism: in the complete detection system, target DNA specifically activated the trans-cleavage activity of the CRISPR-Cas12a complex, enabling it to rapidly cleave the ssDNA structural domains of Aurora and disrupt its effective conformation, resulting in the disrupted Aurora losing its ability to catalyze 4-MUP, which ultimately manifested as a significant reduction in fluorescence signal. This key experimental finding strongly confirms that the presence of *Salmonella typhimurium* DNA effectively activates the cleavage function of the CRISPR-Cas12a system, enabling highly sensitive detection of target pathogens by inducing significant changes in fluorescence signals.

### 3.3. Optimization of Reaction Conditions

**Figure 3:**
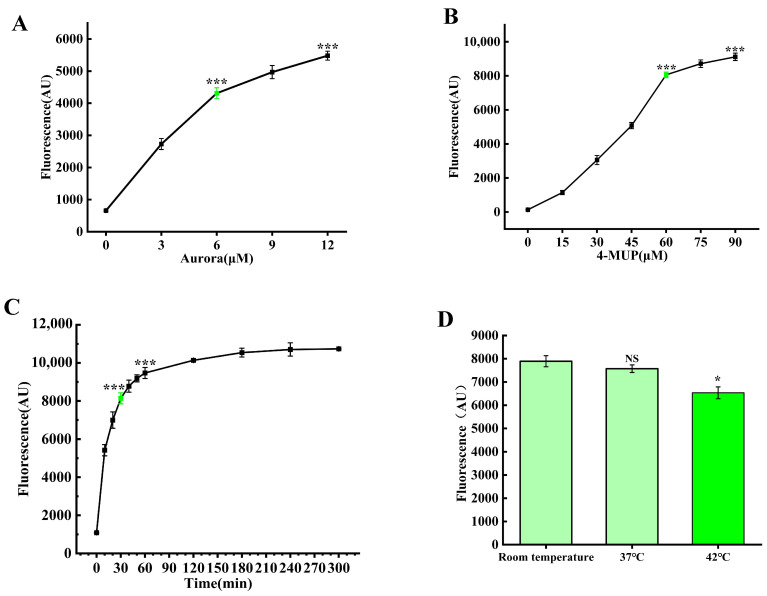
Systematic optimization of critical assay parameters. (**A**) Determination of optimal Aurora enzyme concentration for maximum detection sensitivity. (**B**) Evaluation of 4-MUP substrate concentration effects on signal-to-noise ratio. (**C**) Kinetic analysis of reaction time optimization for Aurora/4-MUP incubation to achieve optimal signal development. (**D**) Temperature-dependent performance assessment of the Aurora/4-MUP reaction system for maximal detection efficiency. *** *p* < 0.001, * *p* < 0.05, NS: not significant (*p* > 0.05), Student’s unpaired *t*-test.

Following successful validation of the label-free fluorescence detection system for *Salmonella typhimurium*, we conducted systematic optimization of critical parameters, including optimal concentration ratios of Aurora enzyme and 4-MUP substrate, reaction duration, and temperature conditions. Through a series of precisely designed experiments and comprehensive data analysis, we established the optimal reaction parameters for this detection system: an Aurora enzyme concentration at 6 μM, a 4-MUP substrate concentration at 60 μM, an optimal incubation period of 30 min, and an optimal reaction temperature at room temperature (Figure 3). The establishment of these optimized conditions provided crucial assurance for the subsequent sensitivity and specificity of the detection system.

### 3.4. Label-Free Detection of Salmonella Using Aurora-Based CRISPR-Cas12a Sensors

**Figure 4:**
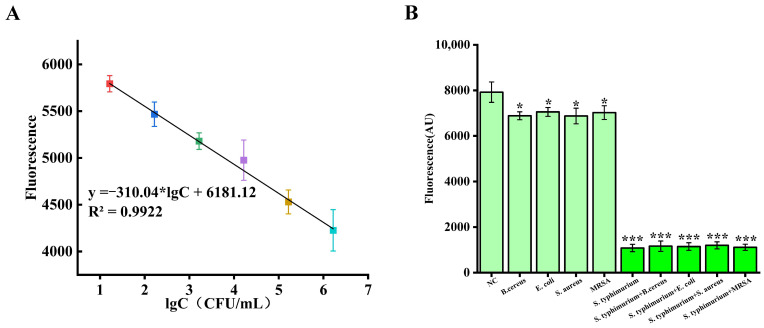
Analytical performance evaluation of *Salmonella typhimurium* detection based on the CRISPR-Cas12a/Aurora system. (**A**) Detection sensitivity linear standard curve demonstrating quantitative detection capability across six orders of magnitude of bacterial concentration. (**B**) Method specificity validation confirming the system’s high selectivity for *Salmonella typhimurium* and robust interference resistance. * *p* < 0.05, *** *p* < 0.001, Student’s unpaired *t*-test.

To comprehensively evaluate the detection sensitivity of the system for *Salmonella typhimurium*, we initially employed national standard methods for precise bacterial enumeration, followed by systematic gradient dilution to establish standardized sample concentrations ranging from 0 to 1.65 × 10^8^ CFU/mL. The RPA-amplified products from these varying concentrations of *Salmonella typhimurium* DNA were subsequently combined with the optimized detection platform, wherein Cas12a, crRNA, and target DNA formed activated ternary complexes exhibiting efficient trans-cleavage activity, with higher DNA concentrations facilitating rapid activation of more Cas12a-crRNA complexes. Figure S2 displays the fluorescence signal responses from different Salmonella sample concentrations, while Figure 4A presents the quantitative relationship between *Salmonella typhimurium* concentration and fluorescence intensity. Through these experimental data, we can clearly observe that as *Salmonella typhimurium* concentration increases, the fluorescence intensity exhibits a systematic decreasing trend. Particularly noteworthy is that within the concentration range of 1.65 × 10^1^–10^6^ CFU/mL, the detection system demonstrates excellent linear response characteristics, with a fitted equation of FI = −310.04 lgC + 6181.12 and a high correlation coefficient of R^2^ = 0.992. Through precise calculation, the system’s limit of detection reaches 1.29 CFU/mL (based on LOD = 3 × SD/slope, where SD represents the standard deviation of the blank control group, and slope represents the gradient of the standard curve). This exceptional sensitivity convincingly demonstrates that the detection platform can reliably detect trace amounts of Salmonella, providing powerful technical support for pathogen detection at ultra-low concentrations in the field of food safety.

Furthermore, we compared the key parameters of different *Salmonella* detection methods, including detection sensitivity, operation time, and practicality (Table 1). Compared to existing *Salmonella* detection methods, our CRISPR-Cas12a/Aurora detection system demonstrates superior analytical performance. With a detection limit of 1.29 CFU/mL, it significantly outperforms traditional PCR methods (10^3^ CFU/mL, 10 h), ELISA (10^3^ CFU/mL, <3 h), and conventional CRISPR-based methods (10^2^ CFU/mL). Additionally, our system completes the entire detection process in just 100 min, substantially faster than traditional PCR methods (10 h) and traditional culture techniques (3–5 days). Further comparison shows that compared with LAMP-based methods (detection limit of 2 × 10^1^ CFU/mL, operation time 45–70 min), our system, while similar in time efficiency, demonstrates significantly improved detection sensitivity. Compared to electrochemical CRISPR sensors (detection limit of 5.5 × 10^1^ CFU/mL, operation time < 2.5 h) and surface-enhanced Raman scattering CRISPR biosensors (detection limit of 110 CFU/mL, operation time 2 h), our method not only performs better in sensitivity but also features a simpler operation process without requiring expensive fluorescently labeled probes. This combination of ultra-high sensitivity and rapid detection time makes our label-free biosensing platform a superior choice for practical applications in food safety monitoring.

To further validate the specificity of the detection system, we performed comparative tests under identical experimental conditions using various potentially interfering bacterial strains, including *Staphylococcus aureus*, *Bacillus cereus*, *Escherichia coli*, and methicillin-resistant *Staphylococcus aureus*, with sterilized PBS serving as a negative control. As shown in Figure 4B, all non-specific bacterial samples produced fluorescence signals approximating background levels (*p* < 0.05), whereas samples containing *Salmonella typhimurium* exhibited fluorescence signals significantly lower than background values (*p* < 0.001). Additionally, we mixed *Salmonella typhimurium* with the competing bacterial strains at a 1:1 ratio and measured their fluorescence values. The results revealed that the fluorescence values of the mixed samples were very similar to those of pure *Salmonella typhimurium* samples, further demonstrating the system’s ability to specifically recognize target bacteria. These results can be attributed to the extremely high affinity and specificity of the Cas12a-crRNA complex, which enables the CRISPR system to accurately recognize and cleave target DNA, thereby effectively differentiating target bacteria from non-target bacteria. These findings strongly confirm the excellent specificity of our developed detection system for *Salmonella typhimurium*.

### 3.5. Application in Real Samples

To comprehensively evaluate the practical utility of our developed CRISPR-Cas12a/Aurora detection system in real-world applications, we strategically selected three representative food matrices with different biochemical compositions for systematic spiking recovery experiments: high-protein matrices (pure milk and chicken) and a high-fiber matrix (lettuce). These food types were specifically chosen due to their microbiological susceptibility and their diverse compositional characteristics that could potentially interfere with nucleic acid-based detection methods.

During the experimental process, *Salmonella typhimurium* DNA at different concentration gradients (0, 1.65 × 10^5^, 1.65 × 10^6^ CFU/mL) was introduced into these three food samples, followed by RPA amplification before incorporation into the optimized detection workflow.

As illustrated in Table 2, the detection results exhibited excellent consistency and accuracy, with recovery rates ranging from 90.91–99.60%, and relative standard deviations (RSDs) maintained between 3.55% and 4.72%. Notably, at both selected concentration levels (1.65 × 10^5^ and 1.65 × 10^6^ CFU/mL), excellent recovery rates were achieved across all three food matrices, demonstrating the method’s outstanding robustness when dealing with different concentrations of Salmonella contamination. Importantly, the blank control samples (with a spiking concentration of 0 CFU/mL) consistently showed “Not detectable” (ND) results across all three food matrices, confirming the absence of false positive signals and establishing the high specificity of our detection system in complex food environments. This clear differentiation between contaminated and uncontaminated samples is particularly important because in actual food safety monitoring, contamination levels may fluctuate, and detection methods need to maintain reliable performance across various concentration gradients while accurately identifying truly negative samples. The consistency exhibited by our system indicates its potential for application in real-world *Salmonella* monitoring scenarios.

The excellent performance across diverse food matrices can be attributed to several factors. First, our optimized sample preparation protocols effectively minimized the influence of inhibitory substances in complex food matrices. Second, the integration of RPA for target amplification, which operates at relatively low temperatures and is less affected by inhibitors compared to PCR, contributes significantly to the method’s robustness. Third, the CRISPR-Cas12a system’s high specificity ensures accurate target recognition even in complex backgrounds. Finally, the Aurora/4-MUP signal generation system exhibits minimal interference from food matrix components, enabling reliable fluorescence measurements. These results indicate that our developed CRISPR-Cas12a/Aurora detection system not only performs excellently under laboratory conditions but also maintains high sensitivity and specificity in actual food samples, providing a promising solution for rapid on-site detection technology in the field of food safety.

## 4. Conclusions

In this study, we successfully established a novel label-free biosensing platform by synergistically integrating CRISPR-Cas12a with Aurora deoxyribozyme. The detection mechanism operates through an elegant molecular cascade: in the presence of target DNA, Cas12a becomes activated and specifically degrades the Aurora enzyme through its trans-cleavage activity, thereby inhibiting Aurora’s ability to catalyze the conversion of 4-MUP to the highly fluorescent product 4-MU. This cascade ultimately enables quantitative pathogen detection through a measurable decrease in fluorescence signal, effectively circumventing the need for traditional dual-labeled fluorescent probes.

Our experimental results demonstrate excellent analytical performance, with the platform achieving a detection limit of 1.29 CFU/mL for *Salmonella typhimurium* and excellent linearity (R^2^ = 0.992) spanning six orders of magnitude (1.65 × 10^1^−10^6^ CFU/mL). The entire detection process from nucleic acid amplification to signal readout can be completed within 100 min, significantly faster than conventional culture-based methods that typically require 3–5 days. Moreover, the system exhibited robust performance in complex food matrices (milk, chicken, and lettuce) with recovery rates ranging from 90.91–99.60% and relative standard deviations between 3.55% and 4.72%, confirming its practical applicability in real-world food safety monitoring scenarios.

This approach offers several distinct advantages: (1) elimination of expensive dual-labeled fluorescent probes, significantly reducing detection costs; (2) utilization of Aurora’s catalytic signal amplification capability, enhancing sensitivity; (3) integration with isothermal amplification technology, simplifying the detection workflow; and (4) modular design that allows for flexible detection of various pathogens through simple replacement of CRISPR guide sequences. These advantages collectively position this biosensing platform as a promising tool for rapid pathogen detection in resource-limited settings.

Nevertheless, it is important to acknowledge that this study is still limited to laboratory settings, and further research is needed to adapt the system for field applications. Future work will focus on developing portable equipment to meet the demand for on-site testing and exploring the potential of a one-pot method that integrates nucleic acid extraction, amplification, and detection into a single reaction vessel, which would further simplify the workflow and enhance the system’s utility for point-of-care diagnostics. Overall, this CRISPR-Cas12a/Aurora cascade provides new insights into pathogen detection technology with applications in food safety, clinical diagnostics, and environmental monitoring.

## Figures and Tables

**Table 1:** Comparison of different methods for the detection of *Salmonella typhimurium*.

Method	LOD (CFU/mL)	DNA Amplification	Time	Samples	References
PCR	1 × 10^3^	PCR	10 h	Raw duck wing	[28]
ELISA	1 × 10^3^	_	<3 h	Milk	[29]
Traditional CRISPR	1×10^2^	PCR	<100 min	Milk	[30]
LAMP	2 × 10^1^	LAMP	45–70 min	Pork	[31]
Electrochemical CRISPR	5.5 × 10^1^	PCR	<2.5 h	Chicken	[7]
CRISPR-SERS biosensor	1.1 × 10^2^	-	2 h	Chicken	[32]
smartphone-read G-quadruplex-based CRISPR-Cas12a bioassay	1	RPA	3 h	Bear and Juice	[33]
BCA-RPA-Cas 12 a	1	RPA	60 min	Milk	[34]
This work	1.29	RPA	100 min	Chicken, Milk, Vegetable	This work

**Table 2:** Detection of spiked *Salmonella typhimurium* in real samples.

Samples	C_(*S. typhimurium*)_ (CFU/mL)	Results (CFU/mL)	Recovery (%)	RSD (%)
Milk	0	ND ^a^	—	—
Milk	1.65 × 10^6^	1.57 × 10^6^	95.15	3.55
Milk	1.65 × 10^5^	1.63 × 10^5^	98.79	3.59
Chicken	0	ND	—	—
Chicken	1.65 × 10^6^	1.49 × 10^6^	93.74	3.60
Chicken	1.65 × 10^5^	1.59 × 10^5^	99.60	4.14
Lettuce	0	ND	—	—
Lettuce	1.65 × 10^6^	1.50 × 10^6^	90.91	4.72
Lettuce	1.65 × 10^5^	1.64 × 10^5^	99.40	3.86

^a^ ND: Not detectable.

## Data Availability

The original contributions presented in the study are included in the article/Supplementary Material, further inquiries can be directed to the corresponding authors.

## Supplementary Materials

The following supporting information can be downloaded at: https://www.mdpi.com/article/10.3390/foods14111892/s1, Figure S1. Schematic diagram of the plate counting method for quantitative detection of bacterial concentration; Figure S2. Scatterplot of bacterial concentrations across eight orders of magnitude; Figure S3. Native-PAGE analysis after Cas12a cleavage of Aurora; Table S1. Nucleic acid sequences used in this experiment.
