# Supplementary material for: CRISPR-Cas12a/Aurora Deoxyribozyme Cascade: A Label-Free Ultrasensitive Platform for Rapid Salmonella Detection
Source: Foods. 2025 May 26;14(11):1892. doi: 10.3390/foods14111892 (PMC12154011; doi:10.3390/foods14111892)
Supplement: Supplementary file 1 [file foods-14-01892-s001.zip › foods-3616779-supplementary.pdf]

CRISPR-Cas12a/Aurora Deoxyribozyme Cascade: A Label-Free Ultrasensitive Platform for Rapid *Salmonella* Detection

Cong Shi [1,2,†], Huimin Tan [1,2,†], Zhou Yu [1,2], Weilin Li [1,2], Yan Man [1,2,*] and Qinghai Zhang [1,*]

[1] Key Laboratory of Environmental Pollution Monitoring and Disease Control, Ministry of Education, School of Public Health, Guizhou Medical University, No. 6 Ankang Road, Guian New Area, Guizhou 561113, China; 15121448276@163.com (C.S.); tanhm3366@163.com (H.T.); yuz2359@163.com (Z.Y.); 18085301797@189.cn (W.L.)

[2] Institute of Quality Standard and Testing Technology, Beijing Academy of Agriculture and Forestry Sciences, Beijing 100097, China

* Correspondence: manyan3669@163.com (Y.M.); zhqh@gmc.edu.cn (Q.Z.)

† These authors contributed equally to this work.

Verification of the Activation Capability of RPA Amplification Products on the CRISPR-Cas12a System

Target DNA from Salmonella typhimurium was added to the CRISPR-Cas12a reaction system to trigger the fluorescent signal, followed by incubation at 37°C for 30 minutes. Subsequently, ThT was introduced and the mixture was further incubated for an additional 30 minutes. For the negative control group, ultrapure water was substituted for target DNA while all other conditions remained identical. Upon completion of the incubation period, fluorescence spectra within the wavelength range of 475-598 nm were measured using an EnVision® Multimode Plate Reader.

Optimization of Aurora Concentration

The primary objective of this study was to determine the optimal concentration ratio of Aurora/4-MUP to achieve maximum fluorescence signal output. We systematically optimized the Aurora concentration and ultimately identified 6 μM as the optimal concentration. The experimental protocol was divided into two critical phases: In the first phase (reaction system A), ultrapure water, crRNA, Cas12a protein, and reaction buffer were combined with Aurora enzyme at various concentration gradients (0-12 μM) and precisely incubated at 37°C for 30 minutes to ensure complete trans-cleavage of Aurora by Cas12a. Subsequently, in the second phase (reaction system B), a mixture containing KCl, $ZnCl_2$, Tris-HCl buffer, DMSO, and 4-MUP (40 μM) was added, followed by further incubation at room temperature for 30 minutes to facilitate complete conversion of the 4-MUP substrate and stable generation of fluorescence signals. The fluorescence intensity was accurately measured using an

EnVision® Multimode Plate Reader.

In principle, one possible strategy to increase fluorescence production is to increase the concentration of Aurora in the reaction system. The significant advantage of this approach is that, unlike simply increasing the substrate concentration, it does not interfere with the background reaction; however, when single-stranded DNA is at a high concentration, it tends to pair with other molecules, resulting in the formation of double-stranded structures that inhibit the normal folding of the functional nucleic acid motifs, interfering with the normal course of the reaction. To ensure high fluorescence production, we investigated the relationship between Aurora concentration and fluorescence changes. We kept the substrate concentration constant and changed only the concentration of Aurora. The experimental results showed that the intensity of fluorescence light production increased linearly from 0 μM to 6 μM, and the reaction rate reached 6.55 ± 0.36 (F/F_0) at 6 μM ($P< 0.001$), and the fluorescence change was not significant when we continued to increase the concentration of Aurora, and the reaction rate was only 8.33 ± 0.48 (F/F_0) at 12 μM ($P< 0.001$) (as shown in Fig. 3A). After analysing the results of the above experiments, we finally selected 6 μM as the optimal working concentration of Aurora. This choice not only ensured that the reaction system could be operated in a highly efficient state, but also showed excellent performance in controlling the experimental cost, which fully demonstrated the practicability and economy of this method in practical applications.

Optimization of 4-MUP Concentration

After determining the optimal Aurora concentration, different gradient

concentrations (0-90 μM) of 4-MUP were added to the system. The optimisation results revealed a clear dose-response relationship: The fluorescence intensity of the system showed an increasing trend with the gradual increase of 4-MUP concentration. The experimental data showed that the reaction rate (61.25±8.53) (F/F_0) ($P<0.001$) was close to its theoretical maximum when the substrate concentration reached the critical point of 60 μM, while further increase in substrate concentration failed to produce a significant enhancement of the fluorescence signals (reaction rate of 69.20±8.82 (F/F_0) at 120 min) ($P<0.001$) (Fig. 3B). This kinetic characteristic may be attributed to enzyme activity inhibition induced by high substrate concentrations, whereby excess substrate molecules reduce catalytic efficiency by altering the enzyme's spatial conformation or molecular structure. Notably, similar substrate inhibition phenomena have been widely reported and verified in various biological enzymatic reaction systems. Based on these experimental results, 60 μM was selected as the optimal working concentration for 4-MUP, which not only ensures high sensitivity of the detection system but also avoids adverse effects from substrate inhibition.

Optimization of Reaction Time

After determining the optimal working concentration of the reaction system, we investigated the time course of the reaction system by establishing a time gradient. The reaction system was incubated at room temperature for 10min, 20min, 30min, 40min, 50min, 60min, 120min, 180min, 240min and 300 min, and the fluorescence intensity of the reaction system was continuously monitored using an EnVision® Multimode

Plate Reader. As shown in Fig. 3C, During the initial phase of the reaction, the fluorescence intensity showed a rapid increasing trend and was very close to the final peak (7.47±0.75) (F/F_0) at about 30 min ($P<0.001$). Although delaying the reaction time enhanced the fluorescence signal, its fluorescence enhancement was limited (8.69 ±0.24 reaction rate at 60 min (F/F_0)) ($P<0.001$). Considering the importance of rapid detection in practical applications, after comprehensive evaluation, we ultimately determined 30 min as the optimal reaction time. This time parameter selection significantly improved detection efficiency while ensuring detection sensitivity, meeting the practical requirements for rapid detection and achieving an optimal balance between reaction time and detection performance.

Optimization of Reaction Temperature

Finally, a systematic temperature gradient experiment was performed in this study to accurately assess the optimal temperature conditions for the Aurora/4-MUP reaction. The reaction system was incubated in different temperature control modules (room temperature, 37°C, 42°C) for 30 min, respectively. As illustrated in Fig. 3D, the experimental data clearly revealed the significant influence of temperature on reaction efficiency: at 25°C, the system generated the strongest fluorescence signal, indicating that the Aurora enzyme exhibits optimal catalytic activity and structural stability at this temperature point; when the temperature was increased to 37° C, the fluorescence intensity tended to decrease slightly, which might mean that the catalytic activity or structural stability of the enzyme was affected to some extent by the increase in

temperature, but this effect was not significant. ($P>0.05$); and when the temperature further increased to 42°C, the fluorescence signal intensity decreased significantly ($P<0.05$). This phenomenon is likely attributed to the disruption of the spatial conformation of Aurora enzyme molecules by high temperature environments, resulting in reduced catalytic efficiency. Based on these experimental results, we determined 25°C (room temperature) as the optimal working temperature for the Aurora-4-MUP reaction system. This parameter selection not only ensures maximum sensitivity of the detection system but also facilitates operation under standard laboratory conditions without requiring additional temperature control equipment, further simplifying the detection process.

Neat 1:10^1 1:10^2 1:10^3 1:10^4 1:10^5 1:10^6 1:10^7 1:10^8 1:10^9

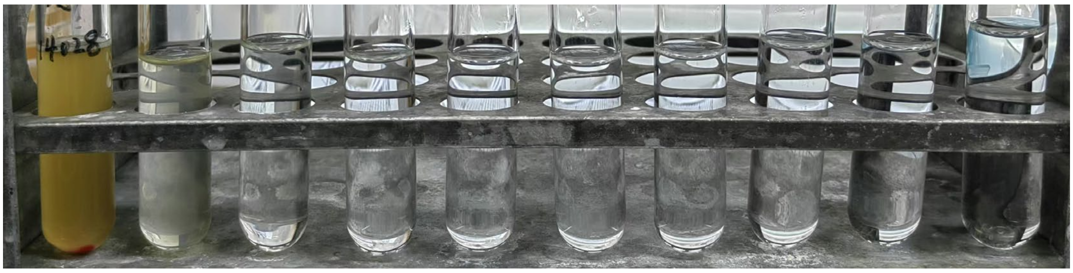

1:10^5 1:10^6

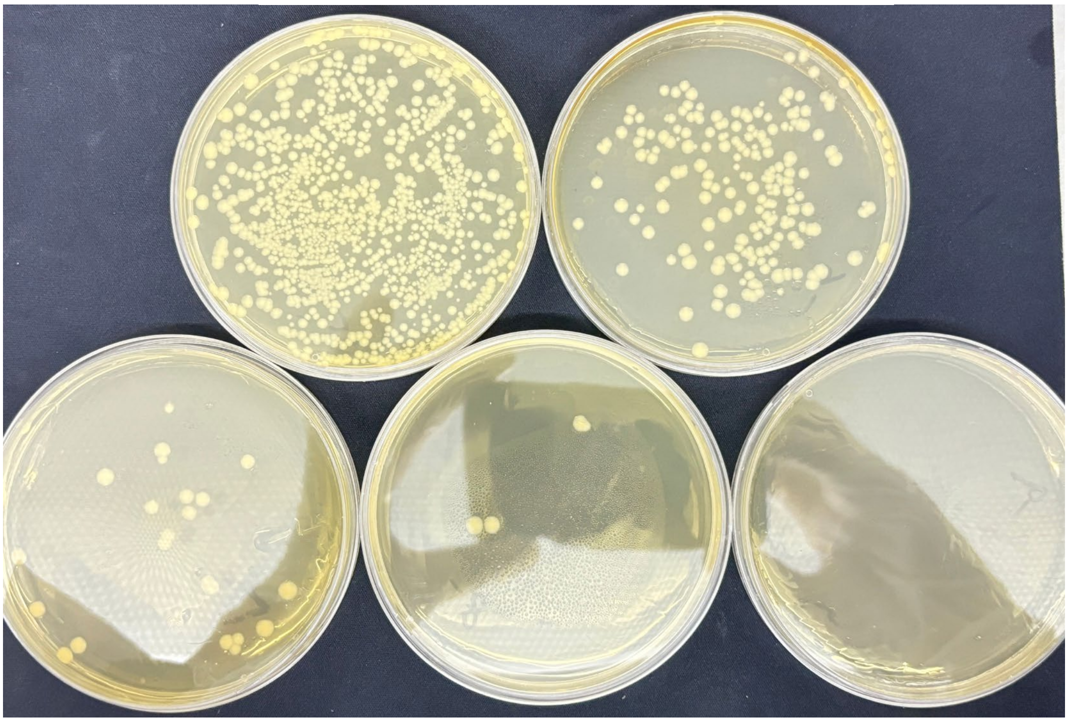

1:10^7 1:10^8 1:10^9

Figure S1. Schematic diagram of the plate counting method for quantitative detection of bacterial concentration.

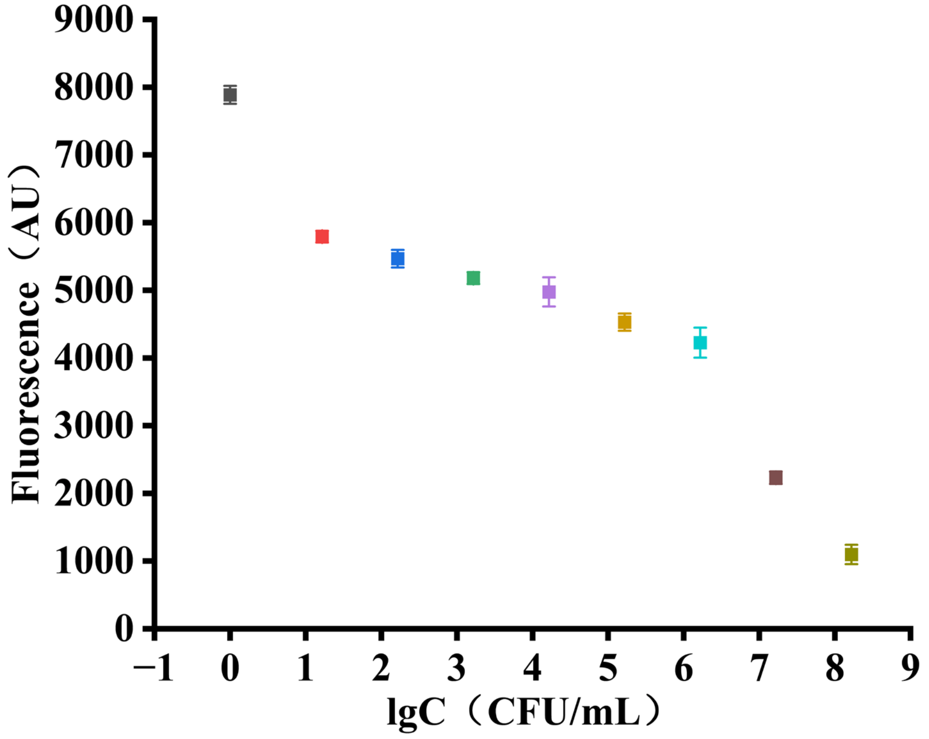

Figure S2. Scatterplot of bacterial concentrations across eight orders of magnitude

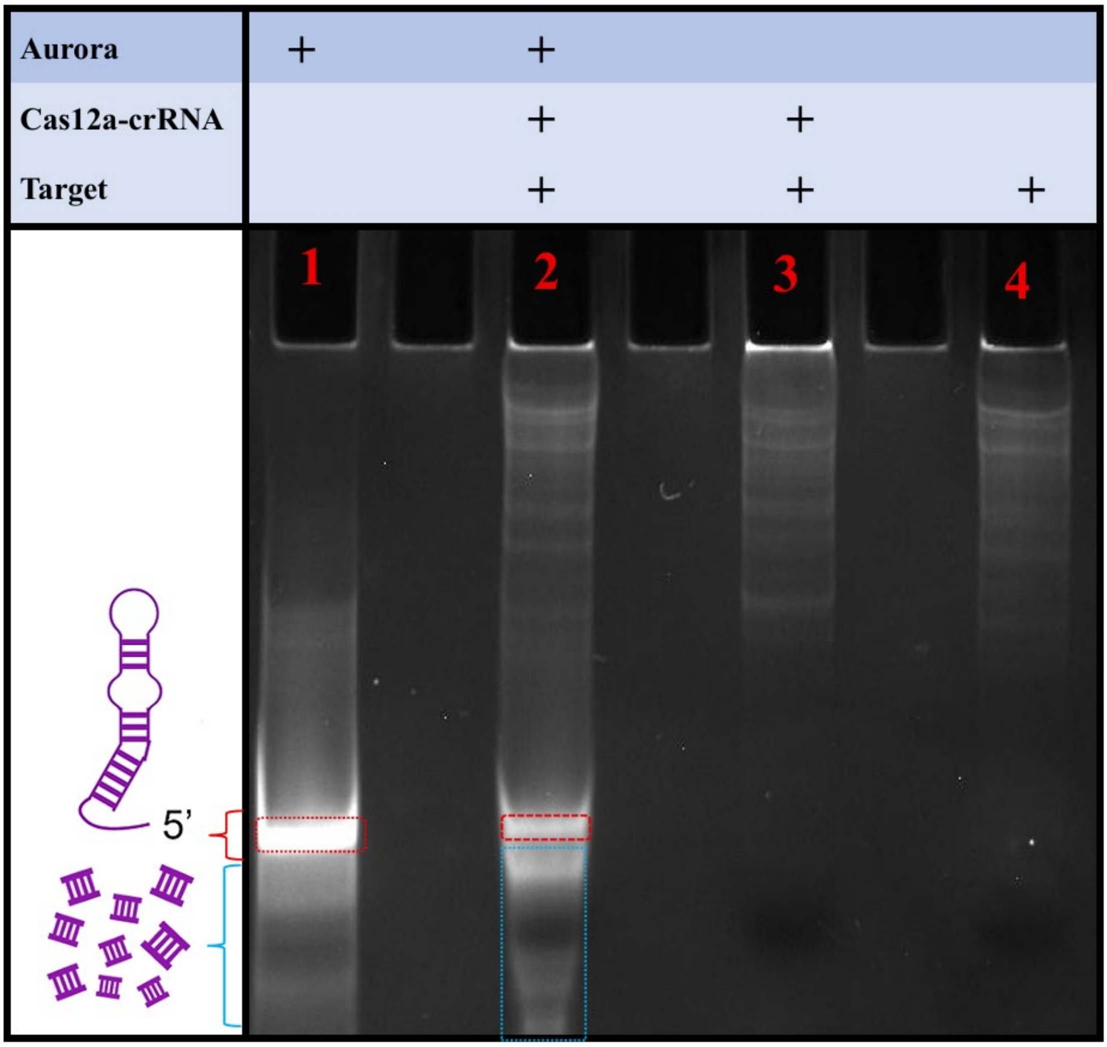

Figure S3. Native-PAGE analysis after Cas12a cleavage of Aurora.

Table S1. Nucleic acid sequences used in this experiment

Name	Sequence
Aurora	GGAAGGGATGACTATGTCCGGTTCCTGTAAGGCATG TGGAGTGTTGT
Salmonella-RPA-F	TGTTGTCTTCTCTATTGTCACCGTGGTCCAG
Salmonella-RPA-R	CATCTGTTTACCGGGCATACCATCCAGAGAAAA
crRNA	UAAUUUCUACUAAGUGUAGAUCCGGGCAUACCAU CCAGAGAAAA
